# Socioeconomic and demographic factors modify the association between informal caregiving and health in the Sandwich Generation

**DOI:** 10.1186/1471-2458-14-362

**Published:** 2014-04-15

**Authors:** Elizabeth K Do, Steven A Cohen, Monique J Brown

**Affiliations:** 1 Virginia Institute of Psychiatric and Behavioral Genetics, Virginia Commonwealth University, Richmond, Virginia, USA; 2 Department of Family Medicine and Population Health, Division of Epidemiology, Virginia Commonwealth University, 830 East Main Street- 8th Floor, Richmond, Virginia 23298, USA; 3 Center for Clinical and Translational Research, Virginia Commonwealth University, Richmond, Virginia, USA

**Keywords:** Informal caregiving, Older population, Race/ethnicity, Income, Children

## Abstract

**Background:**

Nearly 50 million Americans provide informal care to an older relative or friend. Many are members of the “sandwich generation”, providing care for elderly parents and children simultaneously. Although evidence suggests that the negative health consequences of caregiving are more severe for sandwiched caregivers, little is known about how these associations vary by sociodemographic factors.

**Methods:**

We abstracted data from the Behavioral Risk Factor Surveillance System to determine how the association between caregiving and health varies by sociodemographic factors, using ordinal logistic regression with interaction terms and stratification by number of children, income, and race/ethnicity.

**Results:**

The association between informal caregiving and health varied by membership in the “sandwich generation,” income, and race/ethnicity. This association was significant among subjects with one (OR = 1.13, 95% CI [1.04, 1.24]) and two or more children (OR = 1.17, 95% CI = 1.09, 1.26]), but not in those without children (OR = 1.01, 95% CI [0.97, 1.05]). Associations were strongest in those earning $50,000-$75,000 annually, but these income-dependent associations varied by race/ethnicity. In Whites with two or more children, the strongest associations between caregiving and health occurred in lower income individuals. These trends were not observed for Whites without children.

**Conclusions:**

Our findings suggest that the added burden of caregiving for both children and elderly relatives may be impacted by income and race/ethnicity. These differences should be considered when developing culturally appropriate interventions to improve caregiver health and maintain this vital component of the US health care system.

## Background

The United States (US) population aged 65 years and older is expected to increase from 34 million in 2006 [1] to 71 million by the year 2030 [2]. Within this population are a growing number of elderly persons with chronic health conditions who require special social services and/or informal caregiving by family members and friends. Approximately a quarter of US households provide informal caregiving to an older relative or friend [2]. Overall, informal caregiving has resulted in over $350 billion dollars annually in economic savings for the US [3]. However, the maintenance of such responsibility can result in caregiver stress, leading to negative physical and mental health consequences [4-8] including: loss or reduction in employment [9,10] and decreased quality in childcare [11,12] and marital relationships [13].

Caregivers are forced to miss an average of 6.6 days of work annually due to their caregiving responsibilities [14]. More than one-third of caregivers providing care to older adults eventually choose to reduce work hours, or leave the workforce entirely. Relative to men, women are more likely to leave their jobs once they begin providing care. Both of these options have long-term financial implications for caregivers, including an immediate loss of income and/or potential savings [1].

The role of caregiving and resultant caregiver stress varies by race/ethnicity [15,16]. In dementia caregivers, depression is more common among Whites than in African-Americans, and Whites report caregiving as a more stressful activity than other racial/ethnic groups [17]. Also, the relationship between caregiving and specific health outcomes differs by race/ethnicity. In a study of spousal caregivers, the positive association between informal caregiving and cardiovascular disease was evident in Whites, but not for non-Whites [18].

The impacts of informal caregiving are especially evident in the “sandwich generation” [6], the group of individuals who provide simultaneous care to their young or adolescent children and an older family member or friend [19]. According to a 2001 study, two of every five Americans between the ages of 45 and 55 are caregivers to both their parents and children under the age of 21 [17]. A small, but growing body of evidence suggests that members of the sandwich generation may be at higher risk for impaired health behaviors since caregiving reduces the amount of time available for engaging in personal health behaviors. However, few studies have investigated the association between caregiving and health, comparing caregivers of older adults alone to caregivers of both older adults and children.

Therefore, the objective of this study is to determine how the association between caregiving and caregiver health varies by whether or not the caregiver is also providing care to children, as well as race/ethnicity and socioeconomic status.

## Methods

### Study population

Publicly available data were abstracted from the Behavioral Risk Factor Surveillance System (BRFSS), a federally funded, nationally representative survey of the civilian, non-institutionalized, adult population aged 18 years or older. The CDC and state health departments conduct this survey annually to monitor health-related behaviors and risk factors in the US population. In 2009, all 50 states, plus the District of Columbia, and the three US territories participated in the survey. For each selected household, one adult is randomly identified and interviewed for BRFSS. The median cooperation rate was 75.0% and the median overall response rate was 52.5% [20]. A detailed description of the BRFSS study methodology and survey sampling techniques are available online [21]. The BRFSS study has been approved by Human Research Review Boards from the state departments of health.

To examine the association between caregiving and health, subjects who had missing information on the primary outcome, exposure, and effect modification variables were excluded from analyses.

### Outcome measurement

The main outcome of interest was asked of all 2009 BRFSS participants. Each respondent was asked “Would you say that in general your health is: excellent, very good, good, fair, or poor?” This measure was analyzed as a Likert scale with 1 representing “excellent” and 5 representing “poor” self-reported general health, and has been used in previous studies [22,23].

### Exposure measurements

The primary exposure of interest in the study was caregiving status, as measured by the question previously utilized and tested in prior versions of the BRFSS survey [24,25], “People may provide regular care or assistance to a friend or family member who has a health problem, long-term illness, or disability. During the past month, did you provide any such care or assistance to a friend or family member?” Potential confounders assessed in this analysis included age, sex, BMI, rurality, employment, race/ethnicity, income, and number of children. Age and BMI were analyzed as continuous variables. Rurality was based upon the tertile of population density. Race/ethnicity was based on five calculated categories provided by the BRFSS: Non-Hispanic White, Non-Hispanic Black, Non-Hispanic Asian, Hispanic, and Other. Income was derived from the BRFSS categories. Income was grouped into four categories: <$25,000, $25,000- < $50,000, $50,000- < $75,000, and ≥ $75,000. The number of children under 18 years of age was analyzed as an ordinal variable with three levels: 0, 1, or 2+ children. Race/ethnicity, income, and number of children were also used as effect modification variables to determine if and how the association between caregiving and health varies among sociodemographic groups, the primary objective of the study.

### Statistical analyses

Socioeconomic, demographic, and health characteristics of the study sample were tabulated using mean values of continuous variables and frequencies of categorical variables. Then, ordinal logistic regressions were used to calculate odds ratios (OR) and associated 95% confidence intervals (CI) for the outcome of general health status. ORs above 1 indicate increased odds of reporting poorer general health, and ORs below 1 indicate decreased odds of reporting poorer general health. Both unadjusted and adjusted models were used.

Tests for potential effect modification by number of children, income, and race/ethnicity were conducted, through inclusion of two-way interaction terms and through stratification by number of children, income, and race/ethnicity separately and jointly. Finally, linear trends in the potential effect modification for each of these three factors were tested. Appropriate BRFSS survey weights that account for unequal selection probabilities, non-response, and oversampling were applied for all analyses using PROC SURVEYFREQ in SAS (version 9.3; SAS Institute, Cary, NC) software. This research received no specific grant from any funding agency in the public, commercial or not-for-profit sectors.

## Results

Socioeconomic and demographic characteristics of the analytic sample are displayed in Table 1. From the 440,314 total participants who completed the telephone interview, complete data were available for 292,813 individuals who were included in the final analysis. Of those, 74,135 were self-identified as caregivers and 216,652 were not caregivers. Compared to the overall gender distribution (51.4% female, 48.6% male), a disproportionate amount of females (56.9%) were caregivers compared to males (43.1%). Caregivers had slightly higher BMIs than non-caregivers, and were, on average, one year older than non-caregivers. Non-caregivers were slightly more likely to have had at least one child compared to caregivers. Caregivers were less likely to report excellent or very good health compared to non-caregivers.

**Table 1:** Demographic characteristics of the behavioral risk factor surveillance system sample comparing caregivers to non-caregivers

	**Overall**	**Caregivers**	**Non-caregivers**	**P-value**
N	292813	74135 (24.1)	216652 (75.3)	
Age (years)	46.6 (46.6, 46.7)	47.4 (47.3, 47.5)	46.4 (46.3, 46.5)	< 0.001
Sex				
Females	181449 (51.4)	50163 (56.9)	131286 (49.6)	< 0.001
Males	109338 (48.6)	23972 (43.1)	85366 (50.4)	
BMI (kg/m2)	27.50 (27.48, 27.52)	27.76 (27.71, 27.80)	27.43 (27.40, 27.46)	< 0.001
Type of community				
Rural	97257 (20.2)	25732 (21.6)	71525 (19.8)	< 0.001
Intermediate	97113 (35.2)	24781 (35.7)	72332 (35.0)	
Urban	96417 (44.6)	23622 (42.8)	72795 (45.2)	
Race				
White	231650 (68.9)	58798 (69.8)	172852 (68.6)	< 0.001
Black	25630 (10.5)	7382 (12.3)	18248 (9.9)	
Asian	3614 (3.4)	645 (2.4)	2969 (3.7)	
Hispanic	18065 (13.8)	3914 (11.3)	14151 (14.6)	
Other	9164 (3.5)	2716 (4.2)	6448 (3.2)	
Works for wages				
Yes	144557 (57.2)	38803 (57.1)	105754 (57.3)	< 0.001
No	145577 (42.8)	35185 (42.9)	110392 (42.7)	
Income				
0-25 k	72994 (22.9)	17609 (22.8)	55385 (22.9)	< 0.001
25-50 k	68154 (21.8)	18596 (23.3)	49558 (21.3)	
50-75 k	41242 (14.2)	11127 (14.4)	30115 (14.2)	
75 k+	71809 (29.4)	18532 (28.7)	53277 (29.6)	
Missing	36578 (11.7)	8266 (10.8)	28312 (12.0)	
Children				
None	207042 (56.8)	52990 (59.5)	154052 (56.0)	< 0.001
1	33395 (17.0)	9097 (16.8)	24298 (17.0)	
2+	49951 (26.2)	11959 (23.7)	37792 (27.0)	
Self-reported health				
Excellent	52642 (20.9)	13067 (19.5)	39575 (21.4)	< 0.001
Very good	92757 (33.4)	24117 (32.5)	68640 (33.6)	
Good	87736 (29.8)	23022 (31.3)	64714 (29.3)	
Fair	39087 (11.8)	10073 (12.7)	29014 (11.5)	
Poor	16671 (4.2)	3452 (3.9)	13219 (4.3)	

Poorer health was associated with caregiving, increasing age, male sex, increasing BMI, non-White race/ethnicity, low income, and having no children (Table 2). Examining two-way interactions of race, income, and number of children with caregiving status and their association with self-reported health status, we found that the associations between caregiving and self-reported health varied considerably by number of children. There were statistically significant and fairly consistent interactions showing that compared to individuals without young children, the impact of caregiving on health was more detrimental in individuals with one child under 18 (OR 1.11, 95% CI [1.01, 1.23]). The magnitude of the interaction was even more pronounced in individuals with at least two children (OR 1.18, 95% CI [1.09, 1.28]) in the full model. However, none of the race-caregiving and income-caregiving interactions were statistically significant in these models.

**Table 2:** Parameter estimates for ordinal logistic regression models: unadjusted (Model 1), adjusted for socioeconomic and demographic characteristics (Models 2 and 3), and adjusted for socioeconomic and demographic characteristics with interactions for race/ethnicity (Model 4), income (Model 5), number of children (Model 6), and all three (Model 7)

	**Model 1**	**Model 2**	**Model 3**	**Model 4**	**Model 5**	**Model 6**	**Model 7**
Caregiving	1.11 (1.07, 1.14)	1.07 (1.03, 1.11)	1.07 (1.04, 1.11)	1.08 (1.05, 1.12)	1.07 (1.01, 1.12)	1.01 (0.97, 1.06)	1.01 (0.96, 1.08)
Age (years)		1.01 (1.01, 1.01)	1.02 (1.02, 1.02)	1.02 (1.02, 1.02)	1.02 (1.02, 1.02)	1.02 (1.02, 1.02)	1.02 (1.02, 1.02)
Sex		0.99 (0.96, 1.02)	0.96 (0.94, 0.99)	0.96 (0.94, 0.99)	0.96 (0.94, 0.99)	0.96 (0.94, 0.99)	0.96 (0.49, 0.99)
BMI (kg/m2)		1.09 (1.08, 1.09)	1.08 (1.08, 1.08)	1.08 (1.08, 1.08)	1.08 (1.08, 1.08)	1.08 (1.08, 1.08)	1.08 (1.08, 1.08)
Type of community							
Rural		1.19 (1.15, 1.23)	1.18 (1.14, 1.22)	1.18 (1.14, 1.22)	1.18 (1.14, 1.22)	1.18 (1.14, 1.22)	1.18 (1.14, 1.22)
Intermediate		1.02 (0.99, 1.05)	1.08 (1.05, 1.12)	1.08 (1.05, 1.11)	1.08 (1.05, 1.12)	1.08 (1.05, 1.12)	1.08 (1.05, 1.12)
Works for wages		0.47 (0.45, 0.48)	0.60 (0.58, 0.62)	0.60 (0.58, 0.62)	0.60 (0.58, 0.62)	0.60 (0.58, 0.62)	0.60 (0.58, 0.62)
Race (ref = white)							
Black			1.35 (1.28, 1.42)	1.37 (1.29, 1.45)	1.35 (1.28, 1.42)	1.35 (1.28, 1.42)	1.37 (1.29, 1.46)
Asian			1.37 (1.23, 1.52)	1.35 (1.20, 1.51)	1.37 (1.23, 1.52)	1.37 (1.23, 1.52)	1.35 (1.20, 1.52)
Hispanic			1.69 (1.60, 1.80)	1.74 (1.63, 1.85)	1.69 (1.60, 1.80)	1.70 (1.60, 1.80)	1.76 (1.65, 1.87)
Other			1.36 (1.25, 1.48)	1.33 (1.20, 1.47)	1.36 (1.25, 1.48)	1.37 (1.23, 1.52)	1.33 (1.21, 1.48)
Income (ref = 75 k+)							
0-25 k			3.14 (3.01, 3.28)	3.14 (3.00, 3.28)	3.15 (3.00, 3.30)	3.14 (3.00, 3.28)	3.12 (2.97, 3.27)
25-50 k			1.62 (1.56, 1.69)	1.62 (1.56, 1.69)	1.62 (1.55, 1.69)	1.62 (1.56, 1.68)	1.61 (1.55, 1.68)
50-75 k			1.17 (1.13, 1.22)	1.17 (1.13, 1.22)	1.16 (1.11, 1.22)	1.17 (1.13, 1.22)	1.16 (1.11, 1.21)
Children (ref = none)							
1 child			0.97 (0.92, 1.01)	0.97 (0.92, 1.01)	0.97 (0.92, 1.01)	0.94 (0.90, 0.99)	0.94 (0.89, 0.99)
2 or more children			0.86 (0.82, 0.90)	0.86 (0.82, 0.89)	0.86 (0.82, 0.89)	0.83 (0.79, 0.87)	0.82 (0.79, 0.86)
Race-caregiving intx							
Black x Caregiving				0.96 (0.85, 1.08)			0.94 (0.84, 1.06)
Asian x Caregiving				1.11 (0.84, 1.48)			1.10 (0.83, 1.46)
Hispanic x Caregiving				0.88 (0.75, 1.04)			0.86 (0.73, 1.01)
Other x Caregiving				1.08 (0.89, 1.31)			1.06 (0.87, 1.29)
Income-caregiving intx							
0-25 k x Caregiving					0.99 (0.90, 1.08)		1.02 (0.93, 1.12)
25-50 k x Caregiving					1.00 (0.90, 1.09)		1.02 (0.93, 1.12)
50-75 k x Caregiving					1.03 (0.94, 1.12)		1.03 (0.94, 1.13)
Children-caregiving intx							
1 child x Caregiving						1.10 (1.00, 1.21)	1.11 (1.01, 1.23)
2+ children x Caregiving						1.17 (1.08, 1.26)	1.18 (1.09, 1.28)

### Race/Ethnicity

The association between caregiving and health varied by race/ethnicity. Overall, there was a small, but statistically significant association between caregiving and poor health (OR = 1.07, 95% CI [1.03, 1.11]) (Table 3). Similar results were observed for Whites (OR = 1.10, 95% CI [1.07, 1.14]) and Others (OR = 1.21, 95% CI [1.01, 1.45]). No significant associations were observed for Blacks, Asians, or Hispanics, when considering each racial/ethnic group separately.

**Table 3:** Parameter estimates for adjusted ordinal logistic regression models stratified by race/ethnicity, income, and number of children

		**Overall**	**0**	**1**	**2+**	**p for child trend**
All races	All incomes	1.07 (1.03, 1.11)	1.01 (0.97, 1.05)	1.13 (1.04, 1.24)	1.17 (1.09, 1.26)	0.540
	< 25	1.03 (0.96, 1.11)	0.96 (0.88, 1.04)	0.98 (0.81, 1.17)	1.23 (1.05, 1.47)	0.009
	25-50	1.08 (1.00, 1.16)	1.03 (0.94, 1.12)	1.25 (1.03, 1.51)	1.10 (0.94, 1.29)	0.058
	50-75	1.13 (1.04, 1.22)	1.03 (0.94, 1.13)	1.15 (0.95, 1.39)	1.38 (1.14, 1.66)	0.067
	75+	1.16 (1.09, 1.23)	1.15 (1.05, 1.24)	1.24 (1.07, 1.44)	1.12 (1.01, 1.25)	< 0.001(a)
	P-value for inc trend	< 0.001	< 0.001	< 0.001	< 0.001(a)	
White	All incomes	1.10 (1.07, 1.14)	1.02 (0.98, 1.06)	1.25 (1.14, 1.37)	1.27 (1.18, 1.37)	0.100
	< 25	1.16 (1.08, 1.25)	1.05 (0.97, 1.15)	1.15 (0.93, 1.42)	1.58 (1.28, 1.95)	0.016
	25-50	1.16 (1.09, 1.23)	1.08 (1.01, 1.16)	1.37 (1.12, 1.69)	1.31 (1.11, 1.56)	0.197
	50-75	1.09 (1.01, 1.18)	1.04 (0.94, 1.14)	1.07 (0.86, 1.32)	1.28 (1.07, 1.54)	0.691
	75+	1.12 (1.05, 1.19)	1.09 (1.01, 1.18)	1.29 (1.11, 1.50)	1.07 (0.96, 1.19)	0.011(a)
	P-value for inc trend	0.004(a)	0.191	0.074	< 0.001(a)	
Black	All incomes	1.09 (0.98, 1.22)	0.97 (0.84, 1.12)	1.09 (0.84, 1.40)	1.38 (1.09, 1.74)	0.302
	< 25	0.93 (0.78, 1.11)	0.88 (0.70, 1.10)	1.04 (0.67, 1.62)	1.07 (0.77, 1.47)	0.642
	25-50	1.08 (0.86, 1.35)	1.03 (0.78, 1.37)	0.96 (0.59, 1.57)	1.30 (0.78, 2.17)	0.789
	50-75	1.27 (0.93, 1.75)	0.76 (0.52, 1.01)	1.59 (0.93, 2.73)	2.79 (1.38, 5.62)	0.003
	75+	1.30 (0.97, 1.74)	1.25 (0.81, 1.93)	0.88 (0.49, 1.59)	1.50 (0.90, 2.48)	0.645
	P-value for inc trend	< 0.001	< 0.001	0.395	0.020	
Asian	All incomes	1.17 (0.89, 1.54)	1.07 (0.72, 1.59)	1.24 (0.66, 2.31)	1.32 (0.84, 2.07)	0.969
	< 25	1.20 (0.66, 2.16)	1.24 (0.59, 2.63)	1.10 (0.49, 2.51)	1.35 (0.71, 2.56)	0.724
	25-50	1.28 (0.64, 2.55)	0.87 (0.34, 2.28)	2.38 (0.87, 6.52)	3.23 (1.11, 9.44)	0.040
	50-75	1.33 (0.54, 3.27)	0.97 (0.35, 2.74)	0.75 (0.08, 7.31)	3.23 (0.55, 19.14)	0.207
	75+	1.31 (0.90, 1.92)	1.43 (0.82, 2.50)	1.26 (0.62, 2.56)	1.29 (0.71, 2.34)	0.403
	P-value for inc trend	0.933	0.100	0.240	0.070	
Hispanic	All incomes	0.91 (0.78, 1.06)	0.92 (0.72, 1.18)	0.82 (0.61, 1.10)	1.00 (0.81, 1.24)	0.201
	< 25	0.90 (0.73, 1.11)	0.85 (0.63, 1.15)	0.80 (0.52, 1.23)	1.18 (0.85, 1.63)	0.175
	25-50	0.82 (0.59, 1.15)	0.79 (0.44, 1.42)	1.00 (0.53, 1.88)	0.81 (0.54, 1.23)	0.302
	50-75	1.20 (0.84, 1.73)	1.92 (1.15, 3.23)	0.94 (0.45, 1.96)	0.76 (0.40, 1.43)	0.160
	75+	1.57 (1.11, 2.21)	1.71 (1.08, 2.72)	1.12 (0.50, 2.50)	1.38 (0.85, 2.26)	0.182
	P-value for inc trend	< 0.001	< 0.001	0.579	0.758	
Other	All incomes	1.21 (1.01, 1.45)	0.97 (0.77, 1.22)	1.40 (0.94, 2.08)	1.66 (1.13, 2.44)	0.684
	< 25	1.16 (0.84, 1.60)	0.97 (0.70, 1.36)	0.84 (0.36, 1.94)	1.71 (0.85, 3.46)	0.954
	25-50	1.12 (0.79, 1.60)	0.89 (0.59, 1.34)	2.21 (0.96, 5.06)	1.06 (0.48, 2.35)	0.659
	50-75	1.62 (1.04, 2.52)	0.89 (0.51, 1.54)	1.73 (0.75, 4.03)	4.85 (1.95, 12.04)	0.006
	75+	1.06 (0.76, 1.50)	0.79 (0.47, 1.31)	1.54 (0.70, 3.39)	1.39 (0.74, 2.60)	0.971
	P-value for inc trend	0.489	0.268	0.372	0.357	

(a) Indicates that the linear trend was negative.

### Number of children

In the models simultaneously stratified by income, race/ethnicity, and number of children, the association between self-reported health and informal caregiving varied substantially by these three sociodemographic factors. Examining the subgroup of subjects with no children under age 18 in the household, the association between caregiving and health was not significant (OR = 1.01, 95% CI [0.97, 1.05]). However, the association between caregiving and health was statistically significant in subjects with one (OR = 1.13, 95% CI [1.04, 1.24]) or two or more children (OR = 1.17, 95% CI = 1.09, 1.26]). However, the trend with increased number of children was not significant. In individuals earning less than $25,000 per year, there was a significant positive trend in the association between caregiving and health by increasing numbers of children (p < 0.009). For those earning above $75,000 per year, the association between caregiving and health was statistically significant for the entire subgroup, regardless of the number of children. Similar results were observed in Whites. For Blacks, the association between caregiving and health increased as the number of children in the family increased, but the trend was significant only in those earning between $50,000 and $75,000 annually (p = 0.003).

### Income

Considering all races and family sizes combined, as income increased, the magnitude of the association between caregiving and health increased, as well (p < 0.001). When stratified by race/ethnicity, this positive trend indicating an increasing association of caregiving and health as income increased was present in Hispanics (p < 0.001) and also in Blacks, though marginally significant (p = 0.064). For Whites, there was actually a significant negative linear trend in income as a modifier of the association between caregiving and health (p < 0.001); indicating that as income increased, the association between caregiving and health decreased. The observed trend was small, but statistically significant.

For the entire sample, when stratified by number of children, income was a positive effect modifier of the association between caregiving and health in people without children or with one child (p < 0.001 for both groups). The opposite occurred in people with two or more children; for those subjects, as income increased, the association between caregiving and poor health actually decreased (p < 0.001).

## Discussion

The association between caregiving and the self-reported health status of caregivers varied by sociodemographic factors, including number of children present within a household, race/ethnicity, and income. We found that the association between caregiving and poor health generally increased with the number of children present within a household. This finding suggests that there may be increased caregiver stress among the sandwich generation of adults that care for both their elderly family members or friends and underage children simultaneously. Such observations are consistent with past studies [19] suggesting that members of the sandwich generation may be at higher risk for impaired health behaviors compared to caregivers who provide care only for an older adult.

Regarding differences by race/ethnicity in the association between caregiving and health status, there was a statistically significant association between caregiving and health among Whites and Others. This association was not observed for Blacks, Asians, or Hispanics, indicating that cultural differences may exist influencing the association between caregiving and health status. Cultural differences may influence perceptions of caregiving, as they relate to the availability and use of coping strategies by caregivers to manage their situations [26]. For example, other studies examining this association have identified cultural expectations [27], identity [28], family functioning [29], social support [27,30], and spirituality [27] as potential factors influencing perceptions of caregiving. These factors may help to explain some of the observed individual differences in stress response to the caregiving burden [30] and may be interesting topics to explore in future research.

The association between caregiving and health also varied by income. Curiously, the association tended to be strongest in the intermediate income group earning between $50,000 and $75,000 per year and in those earning $75,000 or more, although these findings were not observed for Hispanics. The directionality of the interaction with income changed as the number of children increased for the overall sample. The nonlinear function between health and income has also been observed previously. In one study, stroke rates and income had a non-linear association, with high rates for low and high income groups, and low rates for middle income groups in one study [31].

The overall trends by income were more difficult to detect within the race/ethnicity subgroups. In Whites, the association between caregiving and health was inconsistently significant as income increased. However, for Whites with at least two children, the association between caregiving and poorer health increased significantly (p < 0.001) as income decreased. This finding suggests that, in Whites, caregiving for both elderly relatives and children may be more burdensome in individuals with fewer economic resources than in individuals with higher socioeconomic status. Although the specific reason for the observed trends remains unclear, one potential explanation is that the variability in health among lower income individuals was slightly higher than in those with higher incomes. Those in the lowest quartile of income had approximately 20% higher variability in the health status outcome variable compared to those in the highest income quartile. Therefore, there is the potential to observe a stronger relationship in the lower income groups due to the increased variability of the outcome, which may account for some of the observed trends by income.

An interesting paradox was found, as well. In the models that contained interaction terms (Table 2), the interactions between race and caregiving, and income and caregiving were not statistically significant. However, in the stratified models, there were substantial differences in the association between caregiving and health by both race and income categories. The reasons for this are unclear and require further investigation. Such findings could be due, in part, to the overall magnitude of the effect modification occurring, as well as the observation that the direction and strength of the effect modification by income did not occur uniformly within all race categories, and therefore would not be detected as significant in the interaction models.

### Study limitations and future research directions

Although our study contributes to the growing literature on caregiving burden experienced by the sandwich generation, results should be interpreted with caution, given its limitations. First, the use of cross-sectional data limited the study’s ability to make causal claims regarding the association of caregiving and health status. Second, we only used one measure of self-reported health status as our outcome of interest. Future studies should examine multiple outcomes to allow for a more comprehensive assessment of caregiver health. Third, income thresholds were limited by those provided by the survey questionnaire. Missing data could also bias the observed results somewhat. The relatively small amount of missing data for health status (0.7%), number of children in the households (0.1%), and race/ethnicity (0.9%) should not affect the results meaningfully. However, 12.7% of eligible respondents did not report income. Among those not reporting income, their average self-reported health status was between those in the lowest and second-lowest income categories. Therefore, people who were less healthy were slightly less likely to report income than healthier respondents, which may bias the observed results. Finally, despite having a large sample size, the observed associations between caregiving and health were clinically quite modest in magnitude, although many were statistically significant.

Directions for future research could include other health outcomes, such as emotional, mental, and physical health measures. Additionally, future studies should examine the potential for the observed associations varying by gender. Previous studies have found that female caregivers experience significantly greater strain in family relationships and declines in health than males [32]. Among caregivers to older adults with dementia, females had significantly more caregiver burden than males [33]. Male caregivers to individuals with dementia have a higher desire to institutionalize their relatives with dementia than females when the quality of the relationship between caregiver and recipient is low, but such differences were negligible when quality of the relationship was high [34]. Thus, there is a distinct need to understand the roles, responsibilities, and effects of caregiving, which might also be explained by gender.

Future studies could also examine, among caregivers, how the overall intensity of caregiving, defined by length of time spent caregiving, hours per week caregiving, and specific activities of daily living and instrumental activities of daily living with which the caregiver assists the care recipient, are associated with caregiver burden with respect to health and well-being. These associations could also be assessed by whether or not the caregiver is a “sandwiched” caregiver. Promising new data sets, such as the National Study of Caregiving, a subset of the new National Health and Aging Trends Study (NHATS) [35], could assist in providing detailed data on both the caregiver and the care recipient to better understand these associations.

## Conclusion

Our study suggests that the association between informal caregiving and health varies by whether or not the caregiver is a member of the “sandwich generation”, income, and race/ethnicity. In general, the association between caregiving and health was strongest in those with more children, but there were important racial/ethnic and income-associated differences in these trends. These important sociodemographic differences should be considered when developing culturally-appropriate policies and programs designed to improve the health of caregivers, in efforts to maintain this invaluable part of the health care system in the US.

## Competing interests

The authors declare that they have no competing interests.

## Authors’ contributions

EKD developed the objective of the manuscript and wrote the background and discussion sections. SAC refined the objective of the manuscript, performed the statistical analysis, and wrote the methods and results sections. MJB contributed to the background and discussion sections and edited the entire manuscript. All authors read and approved the final manuscript.

## Pre-publication history

The pre-publication history for this paper can be accessed here:

http://www.biomedcentral.com/1471-2458/14/362/prepub
